# Global fitness profiling of fission yeast deletion strains by barcode sequencing

**DOI:** 10.1186/gb-2010-11-6-r60

**Published:** 2010-06-10

**Authors:** Tian Xu Han, Xing-Ya Xu, Mei-Jun Zhang, Xu Peng, Li-Lin Du

**Affiliations:** 1 National Institute of Biological Sciences, 7 Science Park Road, Zhongguancun Life Science Park, Beijing, 102206, PR China

## Abstract

A genome-wide deletion library is a powerful tool for probing gene functions and one has recently become available for the fission yeast *Schizosaccharomyces pombe*. Here we use deep sequencing to accurately characterize the barcode sequences in the deletion library, thus enabling the quantitative measurement of the fitness of fission yeast deletion strains by barcode sequencing.

## Background

Over the past decade, the availability of whole genome sequences for several major model organisms has spurred the development of many powerful reverse genetics approaches and, as a consequence, brought about dramatic changes to the way gene functions are analyzed. The ultimate reverse genetics tool, whole-genome deletion mutant libraries, were first created for the budding yeast *Saccharomyces cerevisiae *[1,2]. This resource allows all predicted open reading frames in the budding yeast genome to be studied by analyzing the phenotypes of their deletion mutants. Numerous screens have been conducted with the budding yeast deletion libraries to uncover new genes involved in various biological pathways [3]. In addition, new approaches based on the deletion libraries, such as synthetic genetic array analysis, have been developed to map global genetic interaction networks [4]. The utility of the deletion libraries goes even beyond studying gene functions, as profiling drug-sensitive yeast mutants has allowed the targets of therapeutic compounds to be defined [5-8].

The construction of the budding yeast deletion libraries incorporated the ingenious idea of molecular barcodes, which are a pair of 20-nucleotide-long unique DNA sequences flanking each deletion cassette [9]. The two barcodes for each gene are called uptag (barcode upstream of the KanMX marker gene) and dntag (barcode downstream of the KanMX marker gene), respectively. These barcodes revolutionized the way yeast mutants are phenotyped by allowing thousands of mutant strains to be pooled and analyzed together in a highly parallel fashion. The barcodes can be easily amplified by PCR from genomic DNA extracted from the yeast cells in the mutant pool. The amounts of barcode PCR products serve as a quantitative measure of the cell number of each deletion strain in the mutant pool. Traditionally, oligonucleotide microarrays have been used to deconvolute the identity of the strains in the mutant pool and quantify the amount of each barcode PCR product. Recently, deep sequencing was found to perform equally well [10]. Compared to one-by-one screen of individual deletion mutants, barcode-based analyses of pooled mutants significantly improve the throughput of screens, reduce the amount of reagents used, and avoid the problems associated with strain cross-contamination. The most frequently analyzed phenotype of pooled mutants is the growth rates, or fitness, of the mutant strains. Fitness profiling of mutants under hundreds of growth conditions has led to the conclusion that 97% of the genes in the budding yeast genome are required for optimal growth under at least one condition [11]. In addition to phenotyping single-gene mutants, barcode-based analysis has also been used to study gene-gene interactions [12,13].

Besides budding yeast, the only other major eukaryotic model organism in which gene deletion can be carried out with ease is the fission yeast *Schizosaccharomyces pombe*. With its facile genetics, fission yeast has long been a favorite for biologists studying cell cycle control and chromosome dynamics [14,15]. The fission yeast genome contains about 5,000 protein-coding genes, the smallest number among the commonly used eukaryotic model organisms [16]. Comparative genomic analysis showed that around 500 fission yeast genes have no homologs in the budding yeast, but are conserved in other eukaryotic species, including human, apparently due to lineage-specific gene losses that happened during the evolution of *S. cerevisiae *[17]. The recent availability of genome-wide fission yeast deletion libraries has paved the way for global analysis of fission yeast genes, allowing researchers to take full advantage of the differences between the two yeast models [18]. Importantly, the fission yeast deletion libraries have built-in DNA barcodes, similar to the ones used in the budding yeast deletion libraries. The barcode sequences in each strain need to be experimentally characterized as up to 30% of the barcodes in the budding yeast deletion libraries are known to deviate from the original design [10,19]. Here we report a deep sequencing-based characterization of the barcode sequences in the deletion library and describe a fitness-profiling pipeline that allows the analysis of a fission yeast haploid deletion library in pooled cultures by deep sequencing of the DNA barcodes.

## Results

We used two independent deep sequencing approaches to sequence and deduce the 20-mer barcodes in the haploid Bioneer version 1.0 deletion library (Additional files 1 and 2). We obtained at least one unique barcode sequence for 2,560 strains, which represent about 90% of the strains in the library; and for 2,235 strains, both unique uptag and unique dntag sequences were obtained (Additional file 3). A byproduct of our characterization of the barcodes is the identification of certain defects of the deletion library, including duplicated barcodes, misplaced strains, and contaminated wells (Additional files 4, 5, 6, and 7).

**Figure 1:**
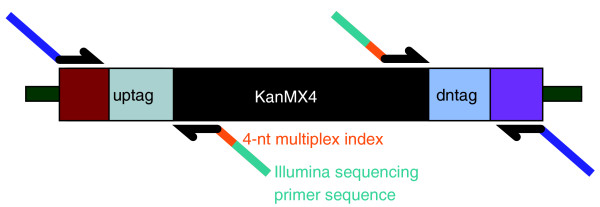
**PCR primer design for barcode sequencing**.

The Illumina Genome Analyzer II sequencing platform can generate over 10 million sequence reads in one sequencing lane. On average, one million reads are sufficient to allow each barcode in a library of 3,000 mutants to be sequenced more than 100 times. To take advantage of the sequencing depth and to reduce the cost of barcode sequencing per screen, we adopted a multiplexing strategy to sequence multiple samples in a single lane. A 4-nucleotide sequence called the multiplex index was incorporated into the PCR primers that harbor the Illumina sequencing primer sequence (Figure 1) [20,21]. Thus, all sequencing reads begin with the index sequences, which allow reads from different samples to be separated. Any two indexes differ by at least two nucleotide substitutions, so that sample misassignment due to sequencing errors is unlikely to happen [22]. Using such multiplex indexes, we routinely combined six-to-nine samples in each sequencing lane. We sequenced the PCR products for 42 sequencing cycles. After parsing the reads into different samples according to their 4-nucleotide index sequences and removing the 18-nucleotide universal primer sequences, the remaining 20-nucleotide sequences were compared to the barcode sequences listed in Additional file 3. Only sequence reads perfectly matching the barcode sequences were kept for further analysis, which typically represented 60 to 70% of the total reads.

**Figure 2:**
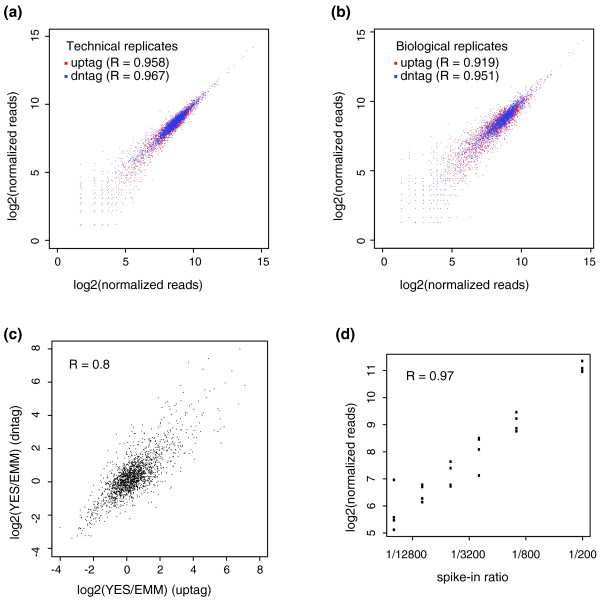
**Reproducibility and linearity of barcode sequencing**. **(a) **Comparison of the barcode sequence read numbers in two technical replicates. Aliquots of the frozen pool of library strains were processed for genomic DNA extraction and barcode PCR in two independent experiments conducted 6 months apart. The barcodes were sequenced in two separate sequencing runs. The sequence read numbers were normalized by total numbers of reads matching either uptags or dntags (listed in Additional file 3). The total matched reads were adjusted to 1 million for uptags or dntags of each sample. Only barcodes with read numbers > 0 in both samples are shown. **(b) **Comparison of barcode sequence read numbers in two biological replicates. Pooled library strains were grown for five generations in rich medium in two independent experiments conducted 6 months apart and the barcodes were sequenced in two separate sequencing runs. The total matched reads were adjusted to 1 million for uptags or dntags of each sample. Only barcodes with read numbers > 0 in both samples are shown. **(c) **Comparison of log ratios of barcode read numbers calculated using uptags and dntags. Pooled mutants grown in rich medium (YES) and minimal medium (EMM) for five generations were used for barcode sequencing analysis. We plot the log ratios of 1,881 strains, which satisfy the condition that read numbers of both uptag and dntag in YES ≥12, and read numbers of both uptag and dntag in EMM > 0. **(d) **The linearity and dynamic range of barcode sequencing assessed using spike-in controls. A *rad32 *deletion strain and a *rad26 *deletion strain from the Bioneer version 1.0 upgrade package (M-1030H-U) were spiked into 24 version 1.0 pooled samples that had been grown in minimal or rich medium for different generations. The ratios between the cell number of each spike-in strain and the total cell number of the version 1.0 pooled strains were 1/200, 1/1,000, 1/2,500, 1/5,000, 1/10,000, and 1/20,000. The read numbers were normalized by total matched reads of the version 1.0 strains. Only uptag reads of the *rad32 *strain are plotted here. See Additional file 8 for the dntag reads of the *rad32 *strain and the barcode reads of the *rad26 *deletion strain.

The barcode sequencing results showed good reproducibility. When two technical replicates were compared, we observed correlation coefficients > 0.95 (Figure 2a). When two independent biological replicates were compared, we observed correlation coefficients > 0.91 (Figure 2b). The presence of two barcodes in each strain allowed the fitness to be assessed by the log ratios of both the uptag and dntag read numbers. When we calculated the log ratios of reads from strains grown in rich medium (yeast extract medium with supplements (YES)) versus minimal medium (Edinburgh minimal medium (EMM)), the values derived from uptags agreed well with those from dntags (Figure 2c). We further evaluated the linearity and dynamic range of barcode sequencing by adding specific amounts of spike-in cells with barcode sequences not in the pooled library. The barcode sequence reads of the spike-in strains showed a linear relationship with the amounts of spike-in cells over two orders of magnitude (Figure 2d; Additional file 8).

As a proof-of-principle test of fitness profiling based on barcode sequencing, we analyzed the growth of deletion mutants in rich medium (YES), minimal medium (EMM), and lysine supplemented minimal medium (EMM+K). We anticipated barcode sequencing to reveal auxotrophic mutants with specific growth defects in the minimal medium. Samples were taken after the mutant pools had grown for one, two, three, four, and five generations in these three types of media. We calculated the fold changes of barcode sequencing read numbers between control condition (YES or EMM+K) and treatment condition (EMM) at multiple time points and combined them into a single value that we called the growth inhibition score (GI), which denotes the level of depletion of the mutants in the treatment condition (see Materials and methods for details of the calculation; Additional files 9 and 10). Mutants that grow normally in both conditions should have GI values around zero, whereas the GI values for auxotrophic mutants are expected to be around 1.

**Figure 3:**
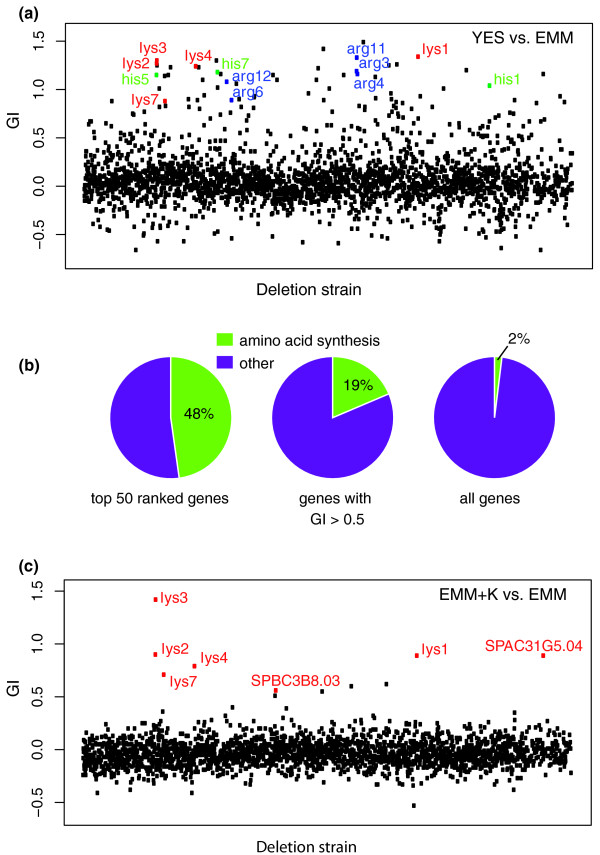
**Auxotrophic mutants were revealed by barcode sequencing**. **(a) **The growth inhibition scores (GI) of the deletion mutants grown in rich medium (YES) versus minimal medium (EMM). The strains are ordered on the x-axis according to their positions in the 96-well plates. There are a total of 19 fission yeast genes in the genome database with three-letter names including *lys*, *arg*, or *his*. A calculated GI value is available for 13 of them. These 13 genes whose mutants are known to be auxotrophic for lysine, arginine, or histidine are highlighted in red, blue, and green, respectively. **(b) **Genes annotated as amino acid biosynthesis genes [GO:0008652] were enriched among the mutants with the highest growth inhibition scores (GI) for YES versus EMM growth conditions. The three pie charts display the percentages of amino acid biosynthesis genes among the genes with the top 50 GI values, among the genes with GI values higher than 0.5, and among all the genes with a GI value. **(c) **The growth inhibition scores (GI) of the deletion mutants grown in lysine supplemented minimal medium (EMM+K) versus minimal medium (EMM). The seven genes annotated as lysine biosynthesis genes [GO:0009085] are highlighted in red.

In Figure 3a we display in a scatter plot the calculated GI values of the mutants grown in rich versus minimal medium (YES versus EMM). The GI values for the majority of the strains fall within -0.5 to 0.5, and the outliers beyond this range are mostly mutants with GI values higher than 0.5. Among these outliers are amino acid auxotrophic mutants, such as the previously known Lys-, Arg-, and His- mutants, which are highlighted in the figure. We applied Gene Ontology (GO) term enrichment analysis to see what types of genes are overrepresented among the genes whose mutants have the highest GI values. Among the top 50 ranked genes, 24 have a GO annotation of amino acid biosynthesis [GO:0008652], which is the ontology term with the highest level of enrichment (24 out of 50, *P*-value = 1.40e-26; Figure 3b). It was previously reported that many fission yeast mutants defective for mitochondrial function can grow in rich medium but cannot grow in EMM medium unless an antioxidant supplement is provided [23,24]. In agreement with previous observations, we found that genes encoding mitochondrial proteins [GO:0005739] were also significantly enriched among the mutants with GI values higher than 0.5 (51 out of 160, *P*-value = 1.90e-08).

Classical fission yeast genetics has isolated lysine auxotrophic mutants corresponding to seven genes, which encode enzymes involved in lysine biosynthesis [25]. Five of them, *lys1*, *lys2*, *lys3*, *lys4*, and *lys7*, have been cloned. In addition, two other genes, *SPAC31G5.04 *and *SPBC3B8.03*, have also been classified by GO annotation as lysine biosynthesis genes based on sequence homology [GO:0009085] [26]. All seven of these genes have corresponding deletion mutants in the Bioneer version 1.0 library. When we calculated the GI values for the EMM+K versus EMM growth conditions, these seven annotated lysine biosynthesis genes were among the top ten with the highest GI values (Figure 3c). The enrichment of expected auxotrophic mutants in the analyses of YES versus EMM and EMM+K versus EMM conditions led us to conclude that barcode sequencing is a sensitive and reliable method for identifying mutants with a significant fitness difference between two growth conditions.

To explore the potential of barcode sequencing in profiling mutants hypersensitive to stress conditions, we decided to examine the fitness changes of the deletion mutants in response to a microtubule depolymerizing drug, thiabendazole (TBZ), and three types of genotoxins: the topoisomerase I inhibitor camptothecin (CPT), the ribonucleotide reductase inhibitor hydroxyurea (HU), and UV irradiation. The modes of action of these four agents are well known and many genes conferring resistance to these agents have been previously characterized, thus allowing us to assess the performance of barcode sequencing-based fitness profiling. To test the reproducibility of barcode sequencing and the use of replicates to reduce the influence of experimental noise, we performed three independent experiments. For two experiments (called A and B) the treatment doses were the same, whereas in the third experiment (called C) the doses were doubled. In each experiment, a pooled mutant culture grown in YES medium was split into five subcultures at the starting time point. Four of them were treated with TBZ, HU, CPT, or UV, and the last one was left untreated as the control. Cell growth was monitored by OD600 and samples for barcode sequencing were collected after the population had doubled five times. Again, a GI value was calculated for each mutant as an indicator of the fitness difference between each pair of control and treatment conditions (Additional file 11).

**Figure 4:**
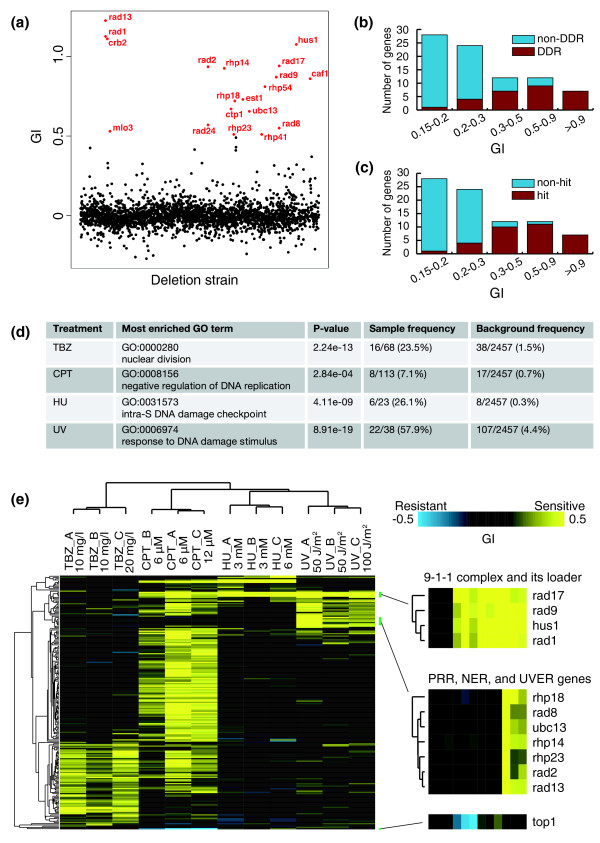
**Profiling of mutants hypersensitive to a microtubule-depolymerizing drug and three genotoxic agents**. The mutant pools grown in YES medium were treated with thiabendazole (TBZ), camptothecin (CPT), hydroxyurea (HU), and UV radiation. Three independent experiments, called A, B, and C, were conducted with an untreated control sample included in each experiment. The treatment doses were the same for experiments A and B, while in experiment C the doses were doubled. **(a) **The growth inhibition scores (GI) of control versus 50 J/m^2 ^UV treatment (experiment UV_A). Strains with GI values > 0.5 are highlighted in red. **(b) **Genes with high GI values in experiment UV_A are more strongly associated with the GO annotation of DNA damage response (DDR) genes. The 83 genes whose GI ≥0.15 in experiment UV_A are classified according to whether they are associated with the GO term 'response to DNA damage stimulus' [GO:0006974]. **(c) **Genes with high GI values in experiment UV_A are more likely to be identified as hypersensitive hits by surpassing the GI and *P*-value cutoffs more than once in three independent experiments. The 83 genes whose GI ≥0.15 in experiment UV_A are classified according to whether they are selected as hypersensitive hits. **(d) **The GO terms most highly enriched among the hypersensitive mutants identified by barcode sequencing. **(e) **Hierarchical clustering analysis of the hypersensitive mutants identified by barcode sequencing. For a detailed view of the heat map, see Additional file 18.

In Figure 4a, GI values of control versus treatment with 50 J/m^2 ^UV in experiment A (UV_A) are displayed in a scatter plot. Most of the mutants with GI values > 0.5 correspond to known DNA damage response (DDR) genes (Figure 4b), reflecting the fact that DDR is one of the most intensively studied areas in fission yeast biology. The percentages of known DDR genes become lower among the genes with GI values between 0.15 and 0.5, even though such GI values still significantly deviate from the average of all GI values (Median + 3 × Normalized interquartile range = 0.14 for the distribution of GI values in UV_A). To reduce false positives due to experimental noise, in addition to a GI value cutoff based on the GI value distribution, we introduced a G-test *P*-value cutoff to remove mutants with less reliable GI values (see Materials and methods for details). Furthermore, we required that in order for a gene to be identified as a hit, its deletion mutant must pass both the GI value filtering and the *P*-value filtering in at least two out of three independent experiments. After applying these filtering steps, only 33 out of the 83 mutants with GI values ≥0.15 in UV_A were eventually identified as UV hypersensitive hits. The percentages of hits in relation to GI values show a similar trend as the percentages of known DDR genes (compare Figure 4c to Figure 4b); namely, mutants with higher GI values are more likely to be selected as hits. Compared to using a cutoff of GI ≥0.15 alone, the percentage of known DDR genes increases from 34% (28 out of 83) to 67% (22 out of 33), a two-fold enrichment. Thus, we conclude that our multi-step filtering scheme based on data from multiple experiments allowed us to distinguish genuinely sensitive mutants, especially the ones with mild sensitivity, from mutants with spuriously high GI values in one experiment due to experimental noise.

Using data from these three experiments and the hit identification criteria described above, we identified 68 TBZ-sensitive mutants, 113 CPT-sensitive mutants, 23 HU-sensitive mutants, and 38 UV-sensitive mutants (Additional files 12, 13, 14, and 15). When GO term enrichment analysis was applied to the hit genes, we found that, as expected, genes involved in nuclear division, a microtubule-mediated process, are heavily enriched among the TBZ-sensitive hits, whereas genes involved in DDR or certain DDR signaling pathways are enriched with the highest statistical significance among the CPT, HU, and UV hits (Figure 4d). We noticed that a number of hit genes not associated with the enriched GO terms do have literature support for their identification as sensitive hits. For example, two genes encoding telomerase subunits, *trt1 *and *est1*, are among the UV-sensitive hit genes. It is known that telomerase mutants become hypersensitive to DNA damage when their chromosomes are circularized [27], an event that probably happened to the telomerase mutants in the deletion library during propagations. A gene encoding the plasma membrane transporter for the vitamin pantothenate, *liz1*, was identified as a HU-sensitive hit in our fitness profiling experiments, consistent with previous reports that *liz1 *mutant cells undergo catastrophic mitosis in the presence of HU [28,29].

A genome-wide screen for fission yeast mutants hypersensitive to DNA damaging agents has recently been reported by Deshpande *et al*. [30]. Different from our barcode-based profiling, Deshpande *et al*. used an earlier version of the Bioneer haploid deletion library (beta version) and performed the screen using a plate-based assay. The mutants of about 2,400 genes exist in both versions of the library and thus the screening results for these mutants should, in theory, be comparable. However, mutants of the same genes in the two libraries may not be identical strains. With this caveat in mind, we compared our screen hits with the Deshpande screen hits for the two treatments both Deshpande *et al*. and we used, CPT and HU (Additional files 16 and 17). Deshpande *et al*. reported 119 CPT-sensitive mutants, 113 of which are present in the version 1.0 library we used. Among these mutants, 102 have at least one barcode decoded by us and 98 have enough sequence reads in the control samples to have GI values calculated in more than one experiment. Thus, 98 out of 119 Deshpande CPT hits are scorable by our barcode sequencing assay. We report here 113 CPT-sensitive hits, 100 of which are present in the beta version library Deshpande *et al*. used. The two CPT hit lists overlap by 47 mutants, which represent 47% of our hits detectable by Deshpande *et al*., and 48% of the Deshpande hits detectable by us. For HU, the two screen hit lists overlap by 11 mutants, which represent 52% of our hits detectable by Deshpande *et al*., and 17% of the Deshpande hits detectable by us. The possible reasons for the discrepancy between the two screening results include the growth condition difference (solid versus liquid medium), different duration of treatment (48 hours versus 5 generations), different treatment doses, and the absence of competition between strains in the plate format versus the presence of competing strains in the pooled screening format. The levels of overlap we see here are similar to the reported overlap (30 to 60%) between solid-medium-based screens and barcode-based pooled mutant screens performed using budding yeast deletion libraries [31].

To reveal patterns of fitness changes in response to TBZ and genotoxin treatments, we applied clustering analysis to the GI values of the 203 hit genes in 12 treatment conditions (Figure 4e; Additional file 18). The dendrogram for the 12 treatment conditions plotted on the horizontal axis indicates that the three types of genotoxic perturbations have a closer relationship to each other than to the microtubule toxin TBZ, consistent with the mechanisms of action of these agents. The three independent experiments for each type of treatment always cluster together, indicating that the barcode sequencing data are reproducible and the two different doses for each type of treatment induced similar fitness changes, at least for most of the sensitive mutants. Within each treatment cluster, experiments A and B did not always cluster together even though the same treatment doses were applied. This is probably due to the fact that experiment A was conducted 5 months earlier than the other two experiments, whereas experiments B and C were carried out in the same week. On the vertical axis, genes whose mutants showed similar patterns of fitness alterations cluster together. As expected, genes grouped together by their fitness profiles often are the ones acting in the same or related biological pathways. For example, as highlighted in Figure 4e, four genes whose mutants showed increased sensitivity to all three types of genotoxins but not to TBZ cluster together and correspond to the genes encoding the proliferating cell nuclear antigen (PCNA)-like checkpoint clamp complex Rad9-Rad1-Hus1 (9-1-1 complex) and the clamp loader protein Rad17 [32]. Another group of genes whose mutants were uniquely sensitive to UV cluster together, and these genes are involved in three UV repair pathways in the fission yeast, namely, postreplication repair, nucleotide excision repair, and the UVDE endonuclease-dependent repair pathway [33,34]. These examples demonstrate that barcode sequencing-based fitness profiling is a promising approach to establishing functional relationships between fission yeast genes.

**Figure 5:**
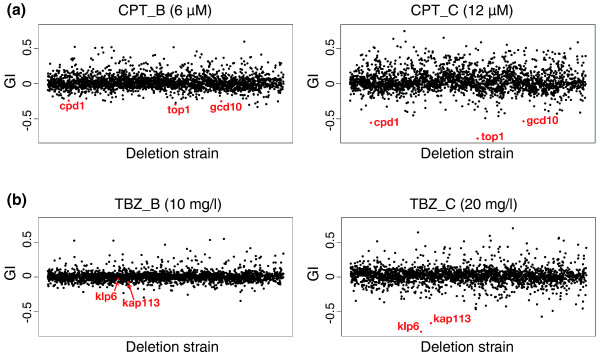
**Camptothecin- and thiabendazole-resistant mutants were revealed by barcode sequencing**. **(a) **The growth inhibition scores (GI) of control versus CPT treatment (experiments CPT_B and CPT_C). Strains with GI values lower than -0.5 in CPT_C are highlighted in red. **(b) **The growth inhibition scores (GI) of control versus TBZ treatment (experiments TBZ_B and TBZ_C). The two strains with lowest GI values in TBZ_C are highlighted in red.

Screening for mutants resistant to a drug may provide unique clues to unveil the mechanism through which the drug acts [35]. However, an extensive budding yeast dataset of barcode-based surveying of bioactive compounds has not been exploited to define truly drug-resistant mutants, presumably due to difficulties in distinguishing true positives from experimental artifacts [11,36]. Thus, it is a welcome surprise that our profiling of CPT- and TBZ-induced fitness changes has allowed *bona fide *drug-resistant mutants to stand out from all the other mutants (Figure 5).

Top1 is the *in vivo *target of CPT and the sensitivity of fission yeast cells to CPT can be completely abolished by a *top1 *mutation [37]. The *top1 *deletion mutant displayed mild sensitivity to HU and was among the 203 hypersensitive hits. Upon inspection of the clustering heat map, we noticed that the *top1 *mutant had GI values below zero in the three CPT treatment experiments (Figure 4e; GI = -0.465 in CPT_A, -0.295 in CPT_B, -0.78 in CPT_C), suggesting that it gained a growth advantage compared to the mutant pool as a whole in the presence of CPT. When the GI values of all mutants were compared, we found that the GI values of the *top1 *mutant were the lowest in experiments CPT_A and CPT_C, and ranked the third lowest in experiment CPT_B (Figure 5a; Additional file 11). Among the three CPT treatment experiments, the higher dose treatment in CPT_C allowed the *top1 *mutant to distinguish itself more from all the other strains with a GI value of -0.78, which corresponds to a roughly 15-fold increase in abundance in the pooled culture after five population doublings. The mutants of two other genes, *cpd1 *and *gcd10*, also displayed conspicuously low GI values in CPT treatments (Figure 5a). These two genes encode the orthologs of the two subunits of a tRNA(1-methyladenosine) methyltransferase in *S. cerevisiae *and human [38,39], suggesting that a defect in tRNA modification may allow cells to become CPT resistant.

Two fission yeast kinesin-8 family proteins, Klp5 and Klp6, are required for normal microtubule dynamics, and disruption of either of their genes leads to hyper-stable microtubules and resistance to TBZ [40,41]. Loss-of-function mutants of *klp5 *and *klp6 *are the most TBZ-resistant fission yeast mutants we could obtain through a transposon-mediated insertional mutagenesis screen for TBZ-resistant mutants (J Li and L-L Du, manuscript in preparation). The mutant of *klp6 *but not *klp5 *is present in the Bioneer deletion library. The GI values of the *klp6 *mutant in the three TBZ treatment experiments were -0.08 for TBZ_A, -0.03 for TBZ_B, and -0.8 for TBZ_C (Figure 5b; Additional file 11). When we ranked the GI values of all mutants from the lowest to the highest, the *klp6 *mutant was ranked number one in TBZ_C, whereas in TBZ_A and TBZ_B it was not among the top 200, suggesting that the *klp6 *mutant grew at rates similar to the mutant pool as a whole in 10 mg/l TBZ, but significantly outpaced other mutants in 20 mg/l TBZ. The second-ranked mutant in TBZ_C is the deletion mutant of *kap113*, which encodes an importin β family protein. An independently made *kap113 *deletion mutant was previously reported to grow better than wild type on YES plates containing 20 mg/l TBZ [42]. Similar to the *klp6 *mutant, in our fitness profiling assays, the *kap113 *mutant only manifested its growth advantage in a higher dose TBZ treatment (Figure 5b).

**Figure 6:**
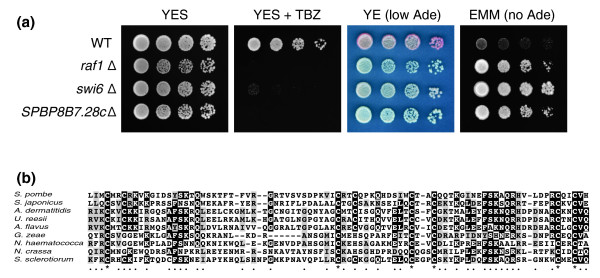
**Barcode sequencing of thiabendazole-treated deletion library led to the identification of a previously uncharacterized gene required for centromere silencing**. **(a) **The deletion mutant of *SPBP8B7.28c *displayed TBZ sensitivity and a centromere silencing defect. Five-fold serial dilution of wild type (WT; DY2776), *raf1Δ *(DY2781), *swi6Δ *(DY2784), and *SPBP8B7.28cΔ *(DY2792) cells were spotted on agar plates of YES medium, YES supplemented with 10 mg/l TBZ, YE medium (*ade6 *mutant colonies turn pink on YE plates due to a low level of adenine), and EMM supplemented with uracil, leucine, and arginine (no adenine). These strains all harbor the *otr1R(SphI)::ade6+ *marker which, when expressed, allows the strains to grow in the absence of adenine and form white colonies on YE plates [50]. **(b) **The protein encoded by *SPBP8B7.28c *shares a conserved domain with proteins from other fungi species. The multiple sequence alignment was created with T-COFFEE [72] and visualized with BOXSHADE 3.21. Six cysteine residues are invariant in the alignment and two FSKxQ motifs are also highly conserved. Accession numbers are [NP_596535.1] (*Schizosaccharomyces pombe*), [XP_002173616.1] (*Schizosaccharomyces japonicus*), [EEQ92506.1] (*Ajellomyces dermatitidis*), [XP_002583495.1] (*Uncinocarpus reesii*), [XP_002379665.1] (*Aspergillus flavus*), [XP_384593.1] (*Gibberella zeae*), [EEU42643.1] (*Nectria haematococca*), [XP_955929.2] (*Neurospora crassa*), [XP_001588826.1] (*Sclerotinia sclerotiorum*).

To our knowledge, no genome-wide screen for TBZ-sensitive fission yeast mutants has been reported until this study; thus, our dataset may offer a unique chance to infer functions of previously unknown genes involved in microtubule organization and chromosome segregation. Fission yeast mutants defective in centromere silencing are known to be hypersensitive to TBZ [43-45], and such mutants were indeed enriched by our screen. Among the 68 genes whose mutants were found to be hypersensitive to TBZ, 9 (*cid12*, *ers1*, *arb1*, *arb2*, *clr4*, *raf1*, *rik1*, *swi6*, and *chp1*) are associated with the GO term 'chromatin silencing at centromere' ([GO:0030702], *P*-value = 2.88e-06) and are involved in the RNA interference (RNAi)-mediated heterochromatin assembly pathway [46]. These genes do not have orthologs in the budding yeast *S. cerevisiae*, which has lost the RNAi machinery during evolution [47,48]. There are ten other genes without apparent *S. cerevisiae *orthologs in our TBZ hypersensitive gene list [17]. We predicted that some, especially those of unknown function, might be involved in centromere silencing. We focused on two genes that are currently annotated as uncharacterized sequence orphans, *SPBP8B7.28c *and *SPBC2G2.14*, whose protein products were shown to be nuclear localized by a genome-wide localization study [49]. The individual Bioneer deletion strains of these two genes were verified by PCR analysis and their TBZ sensitivity confirmed by a plate assay (data not shown). We introduced a centromere silencing marker, *otr1R(SphI)::ade6+*, into these mutants [50]. The mutant of *SPBP8B7.28c *but not *SPBC2G2.14 *failed to silence the expression of the *ade6+ *gene inserted at the centromere *otr *repeat region, indicating that *SPBP8B7.28c *plays an essential role in maintaining normal chromatin state at centromeres (Figure 6a and data not shown). Interestingly, a PSI-BLAST analysis revealed that even though the protein encoded by *SPBP8B7.28c *has no detectable homolog in *S. cerevisiae*, it shares homology with proteins from other fungi species that are known to have RNAi pathways [51] (Figure 6b). A recent paper by Bayne *et al*. [52] (published after this paper was submitted) reported the same phenotypes of the mutant of *SPBP8B7.28c *(named *stc1 *by Bayne *et al*.) and established it as a crucial link between RNAi and heterochromatin formation.

## Discussion

Deep sequencing offers several appealing advantages over microarrays - for example, no need to design and build microarrays, avoidance of the problems associated with cross-hybridization, and potentially more accurate quantification with the 'digital' counts of sequence reads [53]. Thus, it has found wide use in applications previously dominated by microarrays, including fitness profiling of barcoded budding yeast deletion libraries [10]. To fully take advantage of the power of barcode sequencing, it is necessary to accurately sequence the barcodes in the deletion strains, as 20 to 30% of the barcodes in the budding yeast deletion library have been shown to deviate from the original design [10,19]. The barcode sequences we report here are supported by two independent sets of deep sequencing data and have been validated by the fitness profiling assays we conducted. These sequences and the procedures described here should allow any lab with access to a second-generation sequencer to conduct high-throughput barcode-based analysis of fission yeast deletion mutants. The multiplexed sequencing approach reduced the reagent cost of profiling each sample to less than US$100. Different from a recent report on the use of barcode sequencing to analyze budding yeast deletion libraries [10], our multiplexing approach does not require two-step sequencing, and thus the samples can be sequenced exactly the same way as any routine single-end sequencing sample on an Illumina Genome Analyzer. Recently, deep sequencing of transposon-induced mutants has been applied to phenotyping bacteria mutant pools [54-57]. Similar approaches, when developed for fission yeast, may provide an alternative choice to the deletion libraries for functional genomics studies.

We believe that the genome-wide fitness data reported here are useful resources for understanding the functions of many fission yeast genes. In particular, our identification of 203 mutants hypersensitive to TBZ, CPT, HU, or UV based on multiple independent profiling assays has provided phenotypic evidence potentially linking a large number of genes to mitosis and DDR, including many genes without a GO term annotation associating them with these processes. For previously characterized genes, the mutant phenotypes reported here may suggest new aspects of their physiological functions. For previously uncharacterized genes, the barcode-based phenotyping data can be combined with clues provided by other high-throughput methods and comparative genomics to generate hypotheses for follow-up studies, as demonstrated here by the identification of the heterochromatin silencing function of *SPBP8B7.28c*.

Genome-wide budding yeast deletion libraries have been useful for understanding the modes of actions of bioactive chemicals [58]. Even though barcode-based assays in yeast chemical genomics have often focused on detecting drug-sensitive mutants, our data suggest that such assays are equally effective in screening for drug-resistant mutants. The three known CPT-resistant and TBZ-resistant fission yeast mutants displayed dose-dependent growth advantage, suggesting that higher drug doses are better and sometimes required for revealing resistant mutants. Such a requirement may explain why the *top1 *mutant did not behave like a resistant strain when budding yeast deletion mutants treated with CPT at a single dose were analyzed by barcode-based assays [59]. In addition to *top1*, *klp6*, and *kap113*, a number of other mutants also appeared to be resistant to CPT or TBZ based on the GI values we observed in CPT_C and TBZ_C experiments. For example, the low GI values of the tRNA(1-methyladenosine) methyltransferase mutants in the presence of CPT suggested a previously unknown mechanism to achieve cellular resistance to CPT, thus potentially offering new clues to the clinical resistance to Top1-directed anticancer drugs [60,61].

## Conclusions

We have obtained accurate barcode sequences in a haploid fission yeast deletion library and validated them by conducting fitness analysis of barcoded fission yeast deletion strains in pooled cultures. The barcode sequencing data showed good reproducibility and linearity, and we validated the use of barcode sequencing for fitness analysis by detecting auxotrophic mutants that failed to grow in a minimal medium. We applied barcode sequencing to profile the fitness changes of mutants upon treatment with three types of genotoxins and the anti-microtubule compound TBZ. More than 200 mutants hypersensitive to at least one treatment were identified. Genes with known functions in DDR and mitosis were highly enriched among the hypersensitive hits. Unexpectedly, besides sensitive mutants, fitness profiling also revealed mutants resistant to drug treatments, including several mutants resistant to the anticancer drug CPT. Finally, as a demonstration of the use of barcode sequencing in revealing new gene functions, we report the identification of a previously uncharacterized gene required for centromere silencing.

The fission yeast *S. pombe *and the budding yeast *S. cerevisiae *are the two most prominent unicellular eukaryotic model organisms, each contributing greatly to our understandings of many fundamental biological processes [62]. Since their first publication in 1999, the barcoded budding yeast deletion collections have markedly accelerated the pace of discovery in diverse fields that can take advantage of a yeast model [3,63]. We expect that the method we report in this paper will help the barcoded fission yeast deletion collections fulfill their potential and make far-reaching contributions in the coming years.

## Materials and methods

### Media and chemicals

The compositions of YES and EMM media were as described [64]. The genetic background of haploid Bioneer deletion strains is *ura4-D18 leu1-32 ade6-M210 *(or *ade6-M216*); thus, we added uracil, leucine, and adenine to the EMM medium. HU, CPT, and TBZ were from Sigma (St. Louis, MO, USA).

### Construction of a deletion strain pool

Frozen Bioneer version 1.0 haploid library in 96-well plate format (catalog number M-1030H; received on 24 April 2008) was thawed at room temperature and 5-μl portions of the glycerol stock were aspirated from the bottom of the 96-well plates and transferred to deep well plates containing YES agar medium supplemented with 150 mg/l G418 and 100 mg/l carbenicillin. After 2 days of incubation at 30°C, liquid YES medium supplemented with G418 was added and the strains were grown for two more days in a shaker. The liquid cultures were pooled together and briefly centrifuged. The cell pellets were resuspended to a concentration of 15.0 OD600 units per milliliter with fresh liquid YES medium containing Hogness Freezing Medium. The cell suspension was aliquoted into 1.5 ml microtubes at 0.5 ml per tube (7.5 OD600 units) and frozen at -80°C. The recipe for 10× Hogness Freezing Medium stock was: 360 mM K_2_HPO_4_, 132 mM KH_2_PO_4_, 17 mM sodium citrate, 4 mM MgSO_4_, 68 mM (NH_4_)_2_SO_4_, 44% glycerol [65]. The 10× freezing medium was mixed with YES medium at a 1:9 ratio before use.

### Deletion strain pool recovery and growth

Frozen aliquots of the deletion strain pool were thawed at room temperature and washed with YES once, then resuspended in fresh YES liquid medium. The cells were allowed to recover for 5 hours, during which the OD600 increased about 20%. After the recovery period, a sample was harvested and designated as the 0 time point sample. For experiments using EMM medium, cells were collected by centrifugation at the 0 time point and washed with EMM before being transferred into EMM medium. For drug treatment experiments, drugs were added at the 0 time point. For UV treatment, the cells were filtered gently onto the surface of a membrane filter with a pore size of 0.22 μm and then irradiated with UV in a CL-1000 Ultraviolet Crosslinker (UVP, Upland, CA, USA). We monitored the growth of pooled mutant cells by measuring the OD600 of the culture. The cultures were maintained in log phase by diluting with fresh medium when OD600 reached 1.0. For drug treatment experiments, drugs were added to the same concentration into the diluting medium. We harvested 7.5 OD600 units of cells from the cultures after growth for specific numbers of generations.

### Multiplex deep sequencing library preparation

Cells were lysed in TE buffer (10 mM Tris-HCl, 1 mM EDTA, pH 8.0) by beating with glass beads in a FastPrep-24 Instrument (MP Biomedicals, Solon, OH, USA). Genomic DNA was extracted using the MasterPure Yeast DNA Purification Kit (EPICENTRE Biotechnologies, Madison, WI, USA). The barcodes were amplified with Ex Taq HS DNA polymerase (TaKaRa, Otsu, Shiga, Japan) through 30 cycles of 20 s at 94°C, 20 s at 53°C, and 20 s at 72°C. For uptags, the forward primer (upf-X) was 5'-CACGACGCTCTTCCGATCTXXXXGAGGCAAGCTAAGATATC-3', and the reverse primer (upr) was 5'-AGCAGAAGACGGCATACGAGCCTTACTTCGCATTTA-3'. For dntags, the forward primer (dnf-X) was 5'-CACGACGCTCTTCCGATCTXXXXCCAGTGTCGAAAAGTATC-3', and the reverse primer (dnr) was 5'-AGCAGAAGACGGCATACGATTGCGTTGCGTAGG-3'. 'XXXX' in the forward primer sequences denotes the 4-nucleotide multiplex indexes. The PCR products were diluted 200-fold and used as templates for another round of PCR to add sequences needed for Illumina sequencing. The forward primer (seqf) was 5'-AATGATACGGCGACCACCGAGATCTACACTCTTTCCCTACACGACGCTCTTCCGATCT-3', and the reverse primer (seqr) was 5'-CAAGCAGAAGACGGCATACGA-3'. The cycling parameters were: 20 cycles of 20 s at 94°C, 20 s at 56°C, and 20 s at 72°C. The second round PCR products were mixed together in equal molar ratios and gel purified to use as the Illumina sequencing template. Standard single-end sequencing primer was used and 42 cycles of sequencing were carried out with an Illumina Genome Analyzer II. All sequence reads associated with this study have been deposited at the Short Read Archive [SRA012749].

### Barcode sequencing data analysis

The Illumina sequencing reads were assigned to different samples using the 4-nucleotide multiplex index sequences from cycle 1 to cycle 4. The sequences from cycle 5 to cycle 22 were compared to the 18-nucleotide universal primer sequences and only reads with no more than two mismatches were kept. The 20-mer sequences from cycle 23 to cycle 42 were matched with the barcode sequences listed in Additional file 3.

The growth inhibition score (GI) was calculated by:

which is a weighted sum of the quotient of dividing the log fold change by the number of generations. FC_g _is the normalized fold change of read numbers (control versus treatment ratio) at generation g. To avoid dividing by zero, we added a pseudocount of 1 to all reads before calculating the normalized fold change. We required . Mutants whose growth is not inhibited by the treatment will have a growth inhibition score close to 0. The most sensitive mutants, whose cell numbers do not increase at all during the time course, will have a growth inhibition score around 1. To reduce noise caused by low read numbers in the control samples, we did not calculate the growth inhibition scores for barcodes whose read numbers in the control samples were smaller than 12. For a strain with both uptag and dntag sequence reads, if growth inhibition scores were calculated for both barcodes, their averaged value was used for the strain. For a strain with only one barcode having a growth inhibition score, that score was used for the strain.

For calculating the GI values for the samples grown in rich and minimal media for one, two, three, four, and five generations, a_1 _= a_2 _= 0, and a_3 _= a_4 _= a_5 _= 1/3. For the samples treated with TBZ and DNA damaging agents, we collected cells only after five population doublings, thus GI = log_2_FC_5_/5.

To obtain a cutoff GI value for identifying mutants whose growth is inhibited by the treatment conditions, we calculated the median and normalized interquartile range (NIQR) of the distribution of GI values. NIQR = IQR × 0.7413. Median and NIQR are robust statistical estimates of the mean and standard deviation.

To identify mutants hypersensitive to TBZ and DNA damaging agents, for genes with GI values available for only one barcode, we used Median + 3 × NIQR as the GI value cutoff; for genes with GI values available for both uptag and dntag, we used Median + 2.5 × NIQR as the cutoff for the averaged GI values and requested the GI values of both uptag and dntag to be higher than Median + 2 × NIQR. The barcodes with low read numbers in both the control and treatment samples tend to generate unreliable growth inhibition scores. To introduce another cutoff that biases against such barcodes, we calculated the significance values (*P*-values) using the G-test [66], which is a statistical test that does not require replicates and has been successfully used for a number of counting based assays, including quantitative mass spectrometry and serial analysis of gene expression (SAGE) [67-69]. For hit identification of samples treated with TBZ and DNA damaging agents, we requested at least one of the two barcodes associated with a gene to have a *P*-value < 0.005. Finally, we combined the results of the three independent profiling experiments (A, B, and C) by requesting a hit gene to surpass both the GI cutoff and the *P*-value cutoff in at least two of the three experiments.

GO term analyses were conducted with AmiGO version 1.7 using GO database release 2010-01-03 [70].

Hierarchical clustering was carried out with Cluster 3.0, using the correlation (uncentered) similarity metric and the average linkage clustering method. The clustering results were visualized with Java TreeView.

The gene name and gene product functional annotation were obtained from The Sanger Centre *S. pombe *genome database [71]. The ortholog relationship between fission yeast proteins and budding yeast proteins was according to pombe_cerevisiae ortholog table version 2.14 manually curated by V Wood and released on 2 October 2009 [17].

## Abbreviations

CPT: camptothecin; DDR: DNA damage response; EMM: Edinburgh minimal medium; GI: growth inhibition score; GO: Gene Ontology; HU: hydroxyurea; NIQR: normalized interquartile range; RNAi: RNA interference; TBZ: thiabendazole; YES: yeast extract medium with supplements.

## Authors' contributions

TXH performed the smart pooling, constructed the library pools, and carried out barcode-sequencing-based screens. XYX performed the data analysis of barcode sequencing data. MJZ analyzed the paired-end sequencing data. XP generated the paired-end sequencing libraries. L-LD conceived the study, participated in its design and coordination, and drafted the manuscript. All authors read and approved the final manuscript.

## Supplementary Material

Additional file 1 **Information on barcode decoding by deep sequencing**. Click here for file

Additional file 2 **Diagrams of the two methods used to decode barcodes**. **(a) **Paired-end deep sequencing. **(b) **Smart pooling and multiplexed deep sequencing. Click here for file

Additional file 3 **The barcode sequences uniquely associated with the mutants in Bioneer version 1.0 haploid deletion library**. Column A, gene name. Column B, well position according to information provided by Bioneer. Column C, well position annotation: W denotes wrongly placed strains that have been located to a different well by the smart pooling data and PCR analysis (also separately listed in Additional file 5); M denotes the strains that are indicated by deep sequencing to be present in more than one well (also separately listed in Additional file 6); C denotes the wells that are indicated by deep sequencing to be contaminated by a different strain. Column D, uptag sequences. Column E, dntag sequences. Click here for file

Additional file 4 **Barcodes used by more than one deletion strain**. These barcodes cannot be assigned to unique strains and are not included in Additional file 3. Some of the barcodes listed here have been verified by Sanger sequencing (two examples are shown in Additional file 7a). Click here for file

Additional file 5 **Strains whose well positions differ from information provided by Bioneer (annotated with the letter 'W' in Additional file **3A). These strains have all been individually verified by PCR analysis (examples shown in Additional file 7b). Click here for file

Additional file 6 **Strains present in more than one well (annotated with the letter 'M' in Additional file **3). The well positions are predicted by the smart pooling data. The two wells harboring the same strains are often not immediately adjacent wells, and many of them are not even in the same 96-well plates, suggesting that most of the cross-contaminations probably had happened before we received the library from the supplier. Some of the contaminated wells have been verified by PCR analysis (examples shown in Additional file 7b). Click here for file

Additional file 7 **Experimental verification of barcode sequences and strain locations revealed by deep sequencing**. **(a) **Sanger sequencing of deletion cassettes sharing the same barcodes. **(b) **PCR analysis of misplaced strains and those present in more than one well. Click here for file

Additional file 8 **The linearity and dynamic range of barcode sequencing assessed using spike-in controls**. A *rad32 *deletion strain and a *rad26 *deletion strain from the Bioneer version 1.0 upgrade package (M-1030H-U) were spiked into 24 version 1.0 pooled samples that had been grown in minimal or rich medium for different generations. The ratios between the cell number of each spike-in strain and the total cell number of the version 1.0 pooled strains were 1/200, 1/1,000, 1/2,500, 1/5,000, 1/10,000, and 1/20,000. The read numbers were normalized by total matched reads of the version 1.0 strains. **(a) **The normalized read numbers were plotted against the spike-in ratios. **(b) **The observed log fold changes between different spike-in samples were plotted against expected log fold changes. Click here for file

Additional file 9 **The GI values of mutants grown in rich versus minimum medium (YES versus EMM)**. Click here for file

Additional file 10 **The GI values of mutants grown in lysine supplemented minimal medium versus minimum medium (EMM+K versus EMM)**. Click here for file

Additional file 11 **The GI values of mutants treated with TBZ, CPT, HU, and UV**. Click here for file

Additional file 12 **A list of 68 TBZ-sensitive mutants and their GI values**. Click here for file

Additional file 13 **A list of 113 CPT-sensitive mutants and their GI values**. Click here for file

Additional file 14 **A list of 23 HU-sensitive mutants and their GI values**. Click here for file

Additional file 15 **A list of 38 UV-sensitive mutants and their GI values**. Click here for file

Additional file 16 **Comparison of the Deshpande *et al*. CPT screen hits with our profiling results**. Click here for file

Additional file 17 **Comparison of the Deshpande *et al*. HU screen hits with our profiling results**. Click here for file

Additional file 18 **The full heat map of the hierarchical clustering analysis shown in Figure **4e. Click here for file
