# Supplementary material for: Global fitness profiling of fission yeast deletion strains by barcode sequencing
Source: Genome Biol. 2010 Jun 10;11(6):R60. doi: 10.1186/gb-2010-11-6-r60 (PMC2911108; doi:10.1186/gb-2010-11-6-r60)
Supplement: Additional file 1 — Information on barcode decoding by deep sequencing. [file gb-2010-11-6-r60-S1.PDF]

Information on barcode decoding

The Bioneer version 1.0 haploid deletion library (Bioneer catalog number M-1030H) was supplied as glycerol frozen stocks in thirty-one 96-well plates. According to the information provided by Bioneer, there are a total of 2814 strains that correspond to the mutants of 2812 fission yeast genes (two genes each have two corresponding mutants in version 1.0 library: SPBC3B9.11c at wells 4-E10 and 17-F03; SPCC162.12 at wells 11-F09 and 29-D09). We have employed two strategies to obtain the barcode sequences associated with the mutants (Supplementary Figure 1). First, we pooled all the mutants together, extracted the genomic DNA, and used paired-end deep sequencing to acquire both the barcode sequences and their associated genomic DNA sequences flanking the deletion cassette. By mapping the flanking genomic DNA sequences to the annotated genome, we can deduce which gene is disrupted by a deletion cassette harboring a particular barcode (Supplementary Figure 1A). Second, we used a smart pooling design and multiplex deep sequencing to identify the barcodes associated with individual well positions in the 96-well plates (Supplementary Figure 1B). We call the barcode information derived from paired-end sequencing PE data, and the barcode information derived from smart pooling method SP data. PE data link the barcodes with genes but do not provide information on the physical location of any mutants in the library, whereas the SP data link the barcodes with individual mutant strains in the library but cannot by itself ascertain the identity of the mutants. By combining these two approaches, we not only obtained accurate barcode sequences that allows barcode-based high throughput analysis, but also obtained information that facilitates follow-up studies on individual

strains, for example, we have identified dozens of strains whose locations in the library differ from the information given by the supplier (Supplementary Table 3).

Out of the 2812 version 1.0 genes, our PE data analysis identified uptags for 2555 genes and dntags for 2547 genes. Out of the 2814 version 1.0 strains, our SP data analysis identified uptags for 2276 strains and dntags for 2225 strains. That fewer barcodes were deduced from the SP data than the PE data was partly due to dozens of barcodes being used for more than one gene (Supplementary Table 2), and partly due to over a hundred strains existing in more than one well (Supplementary Table 4). These two independent datasets provided corroborating evidence that boosted confidence for assigning barcodes to genes, and we have conservatively used only barcode sequences supported by both PE and SP data for high-throughput phenotypic analysis (Supplementary Table 1).

Paired-end deep sequencing of barcodes

Paired-end sequencing library preparation

The pooled deletion mutant cells were lysed in TE buffer (10 mM Tris-HCl, 1 mM EDTA, pH 8.0) using a FastPrep-24 Instrument (MP Biomedicals). Genomic DNA was extracted from the lysate with the MasterPure Yeast DNA Purification Kit (EPICENTRE Biotechnologies). A primer extension reaction was carried out with Ex Taq HS DNA polymerase (TaKaRa) through 40 cycles of 30 s at 95°C, 15 s at 55°C, and 23 s at 63°C. For uptag sequencing sample preparation, 5’-GGTATTCTGGGCCTCCATGTCG-3’ was used as primer; for dntag sequencing sample preparation, 5’-TGTGAATGCTGGTCGCTATACTG-3’ was used as primer. The primer extension

products were purified with GFX PCR DNA and Gel Band Purification kit (GE Healthcare) and ligated to a linker containing a 6-nt random 3’ overhang. The linker was prepared by annealing two synthetic oligonucleotides: 5’-GGCATTCCTGCTGAACCGCTCTTCCGATCTNNNNNN-NH2-3’, and 5’-P-AGATCGGAAGAGCGGTTCAGCAGGAATGCCGAGACCG-NH2-3’. The ligation reaction products were purified with the GFX kit and used as templates for 30 cycles of PCR amplification. One PCR primer, 5’-CAAGCAGAAGACGGCATACGAGATCGGTCTCGGCATTCCTGCTGA-3’, anneals to the linker sequence. The other PCR primer is a nested primer annealing to the sequences between the barcodes and the primer extension reaction oligonucleotides. For uptags, the nested primer was 5’-AATGATACGGCGACCACCGAGATCTACACTgggtgacccggcggggac-3’; for dntags, the nested primer was 5’-AATGATACGGCGACCACCGAGATCTACACTctgtcgattcgatactaacgcc-3’. These PCR primers contain sequences needed for Illumina paired-end sequencing in their 5’-end. The PCR products were run on an agarose gel and DNA in the size range of 250-300 bp was retrieved from the gel and purified with the GFX kit. The gel-fractionated DNA was amplified for 10 more cycles using the primers 5’-AATGATACGGCGACCACCGA-3’, and 5’-CAAGCAGAAGACGGCATACGA-3’. The amplified DNA was gel purified and used as sequencing template for paired-end sequencing with Genome Analyzer II (Illumina). For uptags, the read 1 sequencing primer was 5’-GGGTGACCCGGCGGGGACGAGGCAAGCT-3’; for dntags, the read 1 sequencing

primer was 5’-CTGTCGATTCGATACTAACGCCGCCATCCAGTGTCGA-3’. Standard Illumina sequencing primer was used for read 2.

Paired-end sequencing data analysis

The 20-nt barcode sequences were extracted from cycle 9 to cycle 28 of read 1 sequences generated by Illumina GAPipeline software. Read 2 sequences from cycle 16 to cycle 42 were aligned to the fission yeast genome with the short oligonucleotide alignment program SOAP [1]. According to the position and orientation of the aligned read 2 sequences, the paired-end reads were assigned to individual protein-coding genes using scripts written in Perl. The barcodes from the paired-end reads assigned to the same gene were then compared with each other and the barcode sequence with the highest read number was chosen as the barcode for the given gene.

Smart pooling and multiplex deep sequencing of barcodes

Smart pool construction

Frozen strain stocks in the Bioneer library were thawed at room temperature and pinned onto YES plate containing 150 mg/l G418 and 100 mg/l carbenicillin using a 96-pin replicator. Two days later, the strains were inoculated into 96-well deep well plates containing liquid YES medium supplemented with G418 and grown for 3 days in a shaker. Cells in each well were dispensed into 5 different pools according to the shifted transversal design of STD(3125;5;5), which can accommodate up to 3125 wells [2]. A total of 25 pools were constructed with each pool containing cells from 563 wells.

Multiplex deep sequencing library preparation

Genomic DNA was extracted from each pool using the MasterPure Yeast DNA Purification Kit (EPICENTRE Biotechnologies). The barcodes in each pool were amplified with Ex Taq HS DNA polymerase through 30 cycles of 20 s at 94°C, 20 s at 53°C, and 20 s at 72°C. For uptags, the forward primer (upf-X) was 5'-CACGACGCTCTTCCGATCTXXXXGAGGCAAGCTAAGATATC-3', the reverse primer (upr) was 5'-AGCAGAAGACGGCATACGAGCCTTACTTCGCATTTA-3'. For dntags, the forward primer (dnf-X) was 5'-CACGACGCTCTTCCGATCTXXXXCCAGTGTCGAAAAGTATC-3', the reverse primer (dnr) was 5'-AGCAGAAGACGGCATACGATTGCGTTGCGTAGG-3'. "XXXX" in the forward primer sequences denotes the 4-digit multiplex indexing tags. Fifty tags were selected from a set of 64 tags that differ from each other by at least two substitutions. The PCR products were diluted 200 folds and used as template for another round of PCR to add sequences needed for Illumina sequencing. The cycling parameters were: 20 cycles of 20 s at 94°C, 20 s at 56°C, and 20 s at 72°C. The same pair of primers was used for all fifty PCR reactions. The forward primer (seqf) was 5'-AATGATACGGCGACCACCGAGATCTACACTCTTTCCCTACACGACGCTCTTCCGATCT-3', the reverse primer (seqr) was 5'-CAAGCAGAAGACGGCATACGA-3'. The second round PCR products were gel purified and mixed together in equal molar ratios to use as the Illumina sequencing template. Standard single-end sequencing primer was used and 42 cycles of sequencing were carried out with Genome Analyzer II.

Smart pooling data analysis

Barcode sequences were extracted from cycle 23 to cycle 42 of the sequencing data. The sequences were assigned to the 25 pools using the multiplex indexing tag sequences from

cycle 1 to cycle 4. Barcodes with total read number lower than 10 were excluded from further consideration. For barcodes appearing in five pools, their well positions were decoded based on the STD(3125;5;5) design [2, 3]. For any barcode appearing in more than 5 pools, the pools were ranked based on the read numbers of the given barcode, and if the read number of the barcode in the sixth-ranked pool was fewer than one tenth of the read number in the fifth-ranked pool, the top five ranked pools were used for well position decoding. When multiple barcodes were assigned to the same well position, they were ranked based on their read numbers, and barcodes with read numbers lower than one-fifth of that of the top ranked barcodes were removed.

References

1. Li R, Li Y, Kristiansen K, Wang J: **SOAP: short oligonucleotide alignment program.** *Bioinformatics (Oxford, England)* 2008, **24:**713-714.
2. Thierry-Mieg N: **A new pooling strategy for high-throughput screening: the Shifted Transversal Design.** *BMC bioinformatics* 2006, **7:**28.
3. Thierry-Mieg N, Bailly G: **Interpool: interpreting smart-pooling results.** *Bioinformatics (Oxford, England)* 2008, **24:**696-703.
