# Supplementary material for: Global fitness profiling of fission yeast deletion strains by barcode sequencing
Source: Genome Biol. 2010 Jun 10;11(6):R60. doi: 10.1186/gb-2010-11-6-r60 (PMC2911108; doi:10.1186/gb-2010-11-6-r60)
Supplement: Additional file 2 — Diagrams of the two methods used to decode barcodes. (a) Paired-end deep sequencing. (b) Smart pooling and multiplexed deep sequencing. [file gb-2010-11-6-r60-S2.PDF]

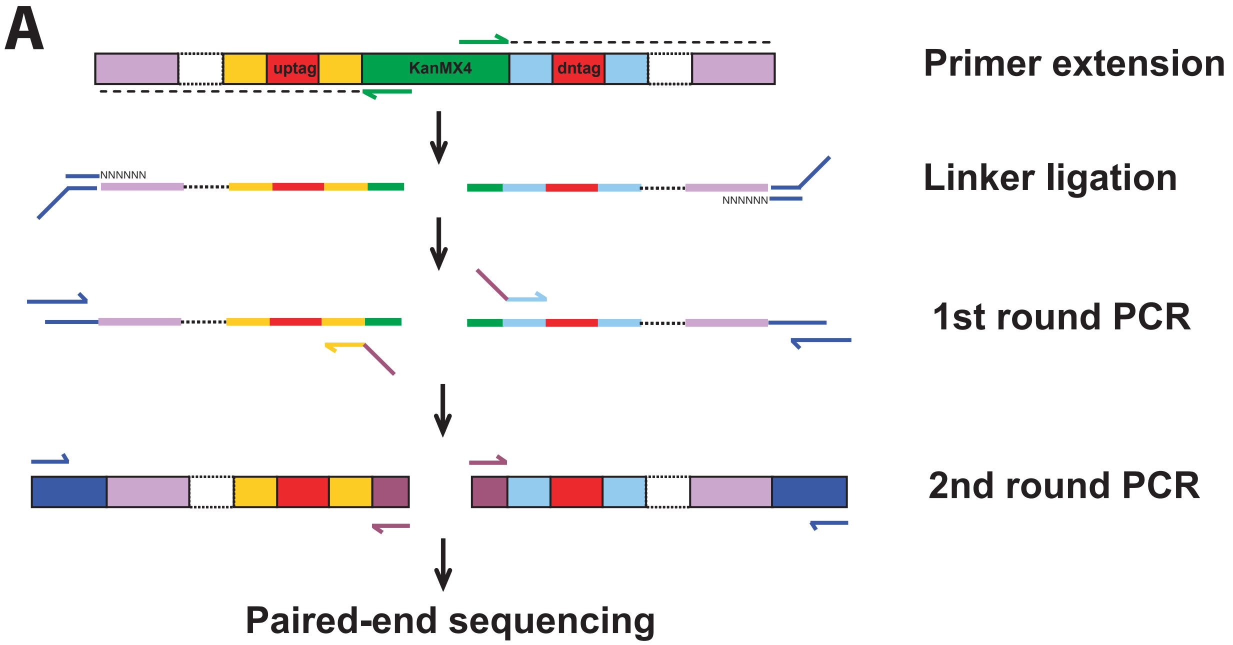


**B**

| Strain | pools | | | | |
|---|---|---|---|---|---|
| Strain 1 | 1 | 6 | 11 | 16 | 21 |
| Strain 2 | 2 | 7 | 12 | 17 | 22 |
| Strain 3 | 3 | 8 | 13 | 18 | 23 |
| Strain 4 | 4 | 9 | 14 | 19 | 24 |
| Strain 5 | 5 | 10 | 15 | 20 | 25 |
| Strain 6 | 1 | 7 | 13 | 19 | 25 |
| ⋮ | ⋮ | ⋮ | ⋮ | ⋮ | ⋮ |
| Strain 2814 | 4 | 9 | 11 | 16 | 21 |

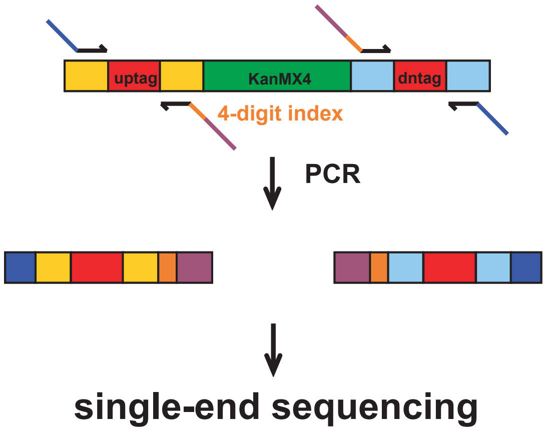


Supplementary Figure 1
Strategies for obtaining the barcode sequences by deep sequencing.
**A**. Paired-end sequencing library contruction procedure.
**B**. Smart-pooling and multiplexed sequencing procedure. The use of four-nucleotide multiplex indexes allowed the PCR products amplified from 25 pools to be sequenced in one sequencing lane.
