# Supplementary material for: Global fitness profiling of fission yeast deletion strains by barcode sequencing
Source: Genome Biol. 2010 Jun 10;11(6):R60. doi: 10.1186/gb-2010-11-6-r60 (PMC2911108; doi:10.1186/gb-2010-11-6-r60)
Supplement: Additional file 7 — Experimental verification of barcode sequences and strain locations revealed by deep sequencing. (a) Sanger sequencing of deletion cassettes sharing the same barcodes. (b) PCR analysis of misplaced strains and those present in more than one well. [file gb-2010-11-6-r60-S7.PDF]

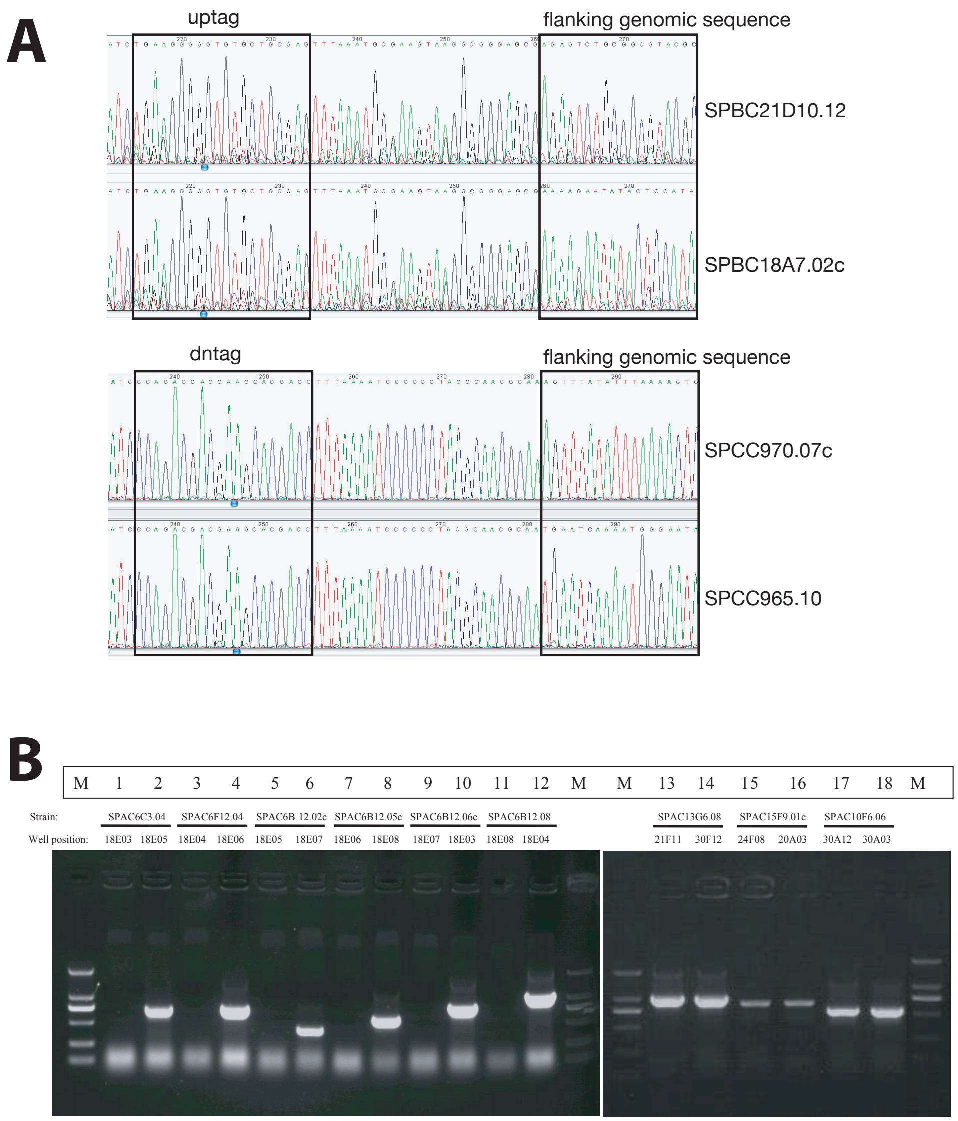

Supplementary Figure 2

A. Sanger sequencing of deletion cassettes sharing the same barcodes.

B. PCR verification of misplaced strains and those present in more than one well.

Left panel. PCR analysis of some misplaced strains. SPAC6C3.04, SPAC6F12.04, SPAC6B12.02c, SPAC6B12.05c, SPAC6B12.06c, and SPAC6B12.08 were not detected in the wells assigned to them by Bioneer (lane 1, 3, 5, 7, 9, and 11, respectively) but were detected in the wells predicted by our analysis based on SP and PE comparison (lane 2, 4, 6, 8, 10, and 12, respectively).

Right panel. PCR analysis of some strains present in more than one well. SPAC13G6.08, SPAC15F9.01c, and SPAC10F6.06 were detected in the wells assigned to them by Bioneer (lane 13, 15, and 17, respectively) and in the wells predicted by our analysis based on SP and PE comparison (lane 14, 16, and 18, respectively).
