# Supplementary material for: Global fitness profiling of fission yeast deletion strains by barcode sequencing
Source: Genome Biol. 2010 Jun 10;11(6):R60. doi: 10.1186/gb-2010-11-6-r60 (PMC2911108; doi:10.1186/gb-2010-11-6-r60)
Supplement: Additional file 8 — The linearity and dynamic range of barcode sequencing assessed using spike-in controls. A rad32 deletion strain and a rad26 deletion strain from the Bioneer version 1.0 upgrade package (M-1030H-U) were spiked into 24 version 1.0 pooled samples that had been grown in minimal or rich medium for different generations. The ratios between the cell number of each spike-in strain and the total cell number of the version 1.0 pooled strains were 1/200, 1/1,000, 1/2,500, 1/5,000, 1/10,000, and 1/20,000. The read numbers were normalized by total matched reads of the version 1.0 strains. (a) The normalized read numbers were plotted against the spike-in ratios. (b) The observed log fold changes between different spike-in samples were plotted against expected log fold changes. [file gb-2010-11-6-r60-S8.PDF]

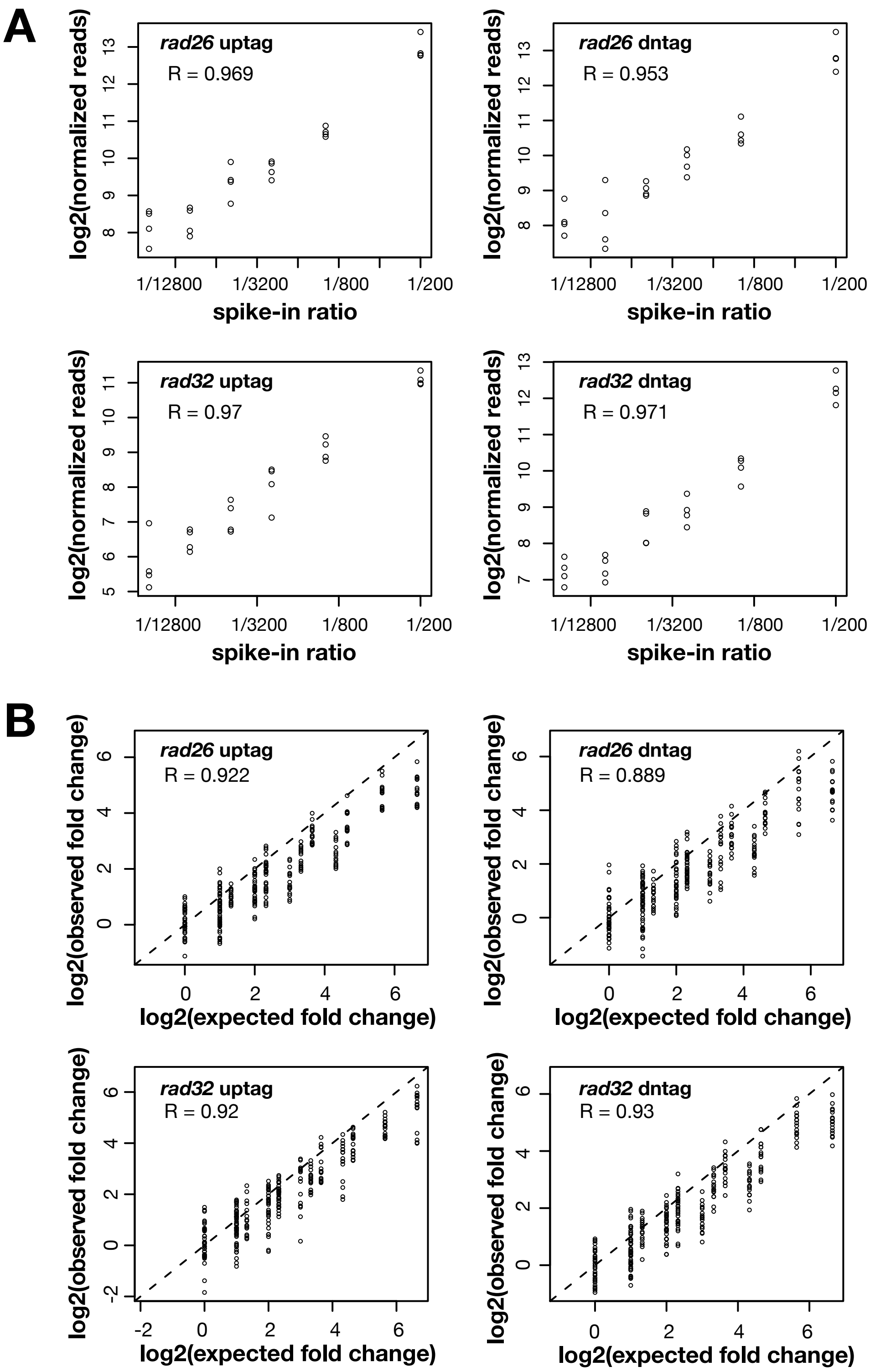

Supplementary Figure 3.

The linearity and dynamic range of barcode sequencing assessed using spike-in controls.

A rad32 deletion strain and a rad26 deletion strain from the the Bioneer version 1.0 upgrade package (M-1030H-U) were spiked into twenty-four version 1.0 pooled samples that had been grown in minimal or rich medium for different generations. The ratios between the cell number of each spike-in strain and the total cell number of the version 1.0 pooled strains were 1/200, 1/1000, 1/2500, 1/5000, 1/10000, and 1/20000. The read numbers were normalized by total matched reads of the version 1.0 strains.

A. The normalized read numbers were plotted against the spike-in ratios.

B. The observed log fold changes between different spike-in samples were plotted against expected log fold changes.
