# Supplementary material for: Global fitness profiling of fission yeast deletion strains by barcode sequencing
Source: Genome Biol. 2010 Jun 10;11(6):R60. doi: 10.1186/gb-2010-11-6-r60 (PMC2911108; doi:10.1186/gb-2010-11-6-r60)
Supplement: Additional file 18 — The full heat map of the hierarchical clustering analysis shown in Figure 4e. [file gb-2010-11-6-r60-S18.PDF]

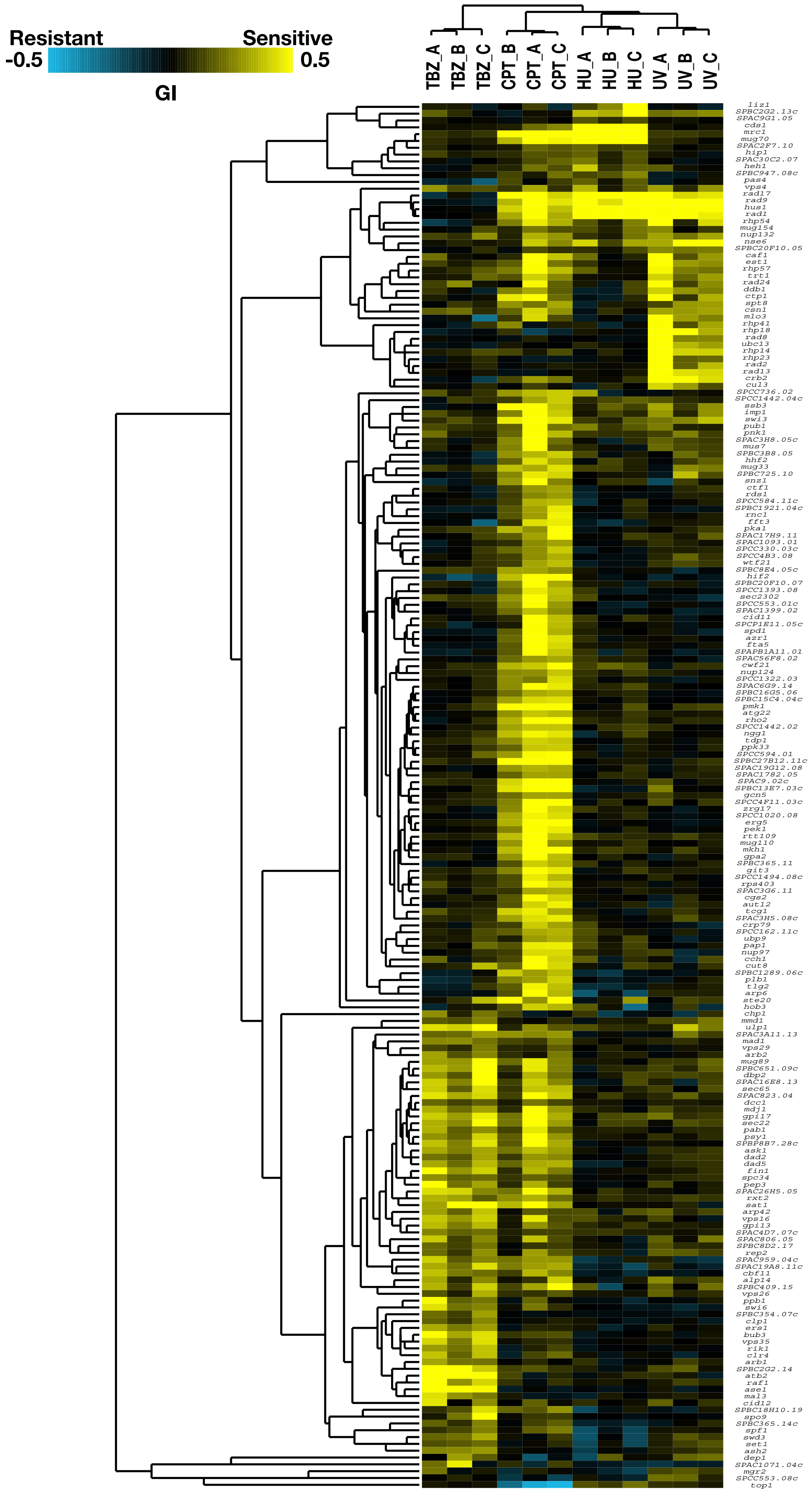

Supplementary Figure 4. The full heat map of the hierarchical clustering analysis shown in Figure 4E.
